# Gender and Vascular Complications in the JAK2 V617F-Positive Myeloproliferative Neoplasms

**DOI:** 10.1155/2011/874146

**Published:** 2011-07-24

**Authors:** Brady L. Stein, Alfred Rademaker, Jerry L. Spivak, Alison R. Moliterno

**Affiliations:** ^1^Division of Hematology/Oncology, Department of Medicine, Feinberg School of Medicine, Northwestern University, Chicago, IL, USA; ^2^Biostatistics Core, Robert H. Lurie Comprehensive Cancer Center, Feinberg School of Medicine, Northwestern University, Chicago, IL, USA; ^3^Division of Hematology, Johns Hopkins University School of Medicine, Baltimore, MD, USA

## Abstract

We previously found that gender influenced the JAK2 V617F allele burden, but it is unknown whether this gender difference in molecular epidemiology influences complications in the myeloproliferative neoplasms (MPNs). Historically, vascular complications represented the most common cause of mortality in polycythemia vera and essential thrombocytosis and contributed to morbidity in primary myelofibrosis. To determine the influence of gender on vascular complications, we retrospectively analyzed associations between gender and vascular complications. Despite their younger age, less prevalent dyslipidemia or smoking history, lower white blood counts, and lower JAK2 V617F allele burden, women had higher rates of abdominal venous thrombosis and comparable rates of all vascular complications. Vascular risk is currently not easily stratified by MPN-disease burden or traditional risk factors. Our analysis contributes to growing literature emphasizing gender differences in the MPN and further supports the important impact of individual and host variation on MPN clinical manifestations, and especially vascular risk.

## 1. Introduction

The myeloproliferative neoplasms (MPNs), essential thrombocytosis (ET), polycythemia vera (PV), and primary myelofibrosis (PMF) share the same acquired genetic lesion, JAK2 V617F, which may explain shared tendencies towards cardinal complications, which include thrombosis, extramedullary hematopoiesis, evolution between phenotypes, and leukemic transformation. However, clinical and pathologic differences among the three disorders raise the question as to how a shared genetic lesion could result in three distinct phenotypes. This question led us to explore the role of the unique host as a modifier of disease pathogenesis. We had previously observed gender differences in disease distribution, including an overrepresentation of women among those with ET and PV, and an underrepresentation of women among those with PMF [1]; also, we found that similar to historical data [2], PV was manifest at a younger age in women compared to men in our cohort. Therefore, we identified gender as a potential host modifier of disease pathogenesis and evaluated whether there were also differences in the JAK2 V617F allele burden between men and women. We found that gender influenced the JAK2 V617F allele burden within the disease phenotypes and the magnitude of change in the JAK2 V617F allele burden through the course of the disease duration, with women having lower mutational burdens than men [3]. 

It is unknown whether these gender differences in molecular epidemiology influence cardinal complications in the MPN. In our prior analysis, among those that experienced phenotypic evolution, women were more likely to evolve from ET to PV, but less likely to evolve from ET to MF [3]. Historical differences in the experience of extramedullary hematopoiesis have been reported. For example, it has been suggested that splenomegaly more commonly complicated the course in women with PV, but survival was better compared to men [2]. Historical literature has also suggested that women with myelofibrosis have a longer duration of illness leading up to splenectomy, a lower complication rate, and a higher survival associated with this procedure compared to men [4]. These suggestions of survival differences by gender have not been confirmed, but a recent publication analyzing the National Cancer Institute's Surveillance, Epidemiology, and End Results (SEER) Program and the North American Association of Cancer Registries (NAACCR), suggest a slight 3-year relative survival advantage in women compared to men (81% versus 79%) [5].

In patients with ET or PV, vascular complications represented the most common cause of morbidity and mortality [6]; in PMF, vascular complications were similarly morbid, as the cardiovascular event rate was comparable to that of ET [7]. In the population at large, traditionally, coronary heart disease (CHD), including myocardial infarctions, more commonly affect men, and women lag in incidence of CHD by a decade, though gender differences narrow with age [8]. Overall, women have lower age-adjusted incidence of stroke compared to men, but this is modified by age, and when comparing younger women (45–54 years), stroke risk is increased compared to men [8, 9]. Studies suggest that incident venous thromboembolic events (VTE) are increased in men, as are rates of VTE recurrence [10–12]. To determine the influence of gender on vascular complications in the MPN, we analyzed associations between gender and vascular complication rate and type; we hypothesized that male sex would influence the tendency toward vascular complications. To test this hypothesis, we retrospectively analyzed a cohort of 270 JAK2 V617F-positive patients from the Johns Hopkins Center for the Chronic Myeloproliferative Disorders.

## 2. Methods

The patient cohort included 270 consecutive JAK2 V617F-positive MPN patients with ET, PV, or myelofibrosis (MF), which included post-ET and post-PV myelofibrosis patients, evaluated at the Johns Hopkins Center for the Chronic Myeloproliferative Disorders between May 2005 and January 2009. The diagnoses of PV and ET were based on the Polycythemia Vera Study Group criteria [13, 14]; the diagnosis of PMF was based on Italian Consensus criteria [15, 16]. MF patients with antecedent ET or PV were characterized accordingly as post-ET MF or post-PV MF [16]. All patients gave written consent for venipuncture and allowed their clinical and laboratory data to be recorded in a database for later analysis; the development of this database was approved by the Johns Hopkins University Institutional Review Board.

### 2.1. Outcome and Variable Assessment

This study was retrospective, and data were derived from abstraction of the medical records, using initial consultation notes and visit notes corresponding with the date of JAK2 V617F allele burden analysis. A history of vascular complication was the outcome of interest; events within 5 years preceding, or at any time following an official diagnosis of ET (*n* = 61), PV (*n* = 161), or MF (post-ETMF, post-PVMF, and PMF) (*n* = 48) were included. Vascular complications were characterized as major arterial (cardiac, or neurologic); major venous (deep vein thrombosis/pulmonary embolism (DVT/PE), abdominal venous thrombosis (AVT) (splenic vein, mesenteric vein, portal vein, and hepatic vein thrombosis), and sinus venous thrombosis)), and minor events (transient ischemic attack (TIA); microvascular disturbance (visual change or digital ischemia), and peripheral arterial thrombosis)). When available, the interval of time between MPN diagnosis and the first vascular event was recorded, as well as the age at the first vascular event. Traditional thrombotic risk factors were recorded as binary variables, including hypertension (by antihypertensive use or medical history), dyslipidemia (by cholesterol-lowering agent use or medical history), diabetes mellitus, and smoking history. 

The JAK2 V617F allele burden was measured using an allele-specific, quantitative real-time polymerase chain reaction (PCR) assay sensitive to 5% of either the wild-type or mutant JAK2 allele; intra-assay replicates did not vary more than 5%, as previously described [1]. A homozygous genotype was determined based on a JAK2 V617F allele burden of ≥55%, while those allele burdens <55% were considered to reflect a heterozygous genotype. Disease duration, antiplatelet use (aspirin) or anticoagulant use (warfarin), cytoreductive or other therapy (hydroxyurea, anagrelide, and interferon), spleen size, white blood cell count (WBC), hemoglobin, and platelet count were assessed at the time of JAK2 V617F determination.

### 2.2. Statistical Analysis

Distribution of outcomes and covariates were described by gender: median and inter-quartile range (IQR) for skewed continuous variables, and number (*N*) and percent (%) for categorical variables. The Mann-Whitney Rank Sum test was used for hypothesis testing of gender differences for continuous variables as appropriate. Testing for gender differences in proportions was performed using the *z* test, or the chi-square statistic when appropriate; Fisher's exact test was used when cell counts in the contingency table were small. Data were analyzed using the STATA 10.1 program (STATA Corp., College Station, Tex).

## 3. Results

### 3.1. Baseline Characteristics

The 270-patient cohort included 164 women and 106 men; women were more likely to distribute in the ET (43/164: 26%) and PV phenotypes (104/164: 63%) than MF (17/164: 10%) compared to men who were more likely to distribute in the PV (57/106: 54%) and MF phenotypes (31/106: 29%) than ET (18/106: 17%) (*P* = 0.001). Women were younger at diagnosis, with a median age difference of 6 years (*P* = 0.001). The median disease duration for all patients was 5 years. The prevalence of hypertension and diabetes mellitus was similar between genders, but women were less likely to have a history of dyslipidemia (9% versus 25%; *P* = 0.001) or a history of tobacco use (22% versus 39%; *P* = 0.01). The prevalence of anti-platelet use (aspirin or clopidogrel) and anticoagulation (warfarin) was similar between men and women. Among laboratory parameters (assessed at the time of JAK2 V617F allele burden determination), the median hemoglobin and platelet counts were similar, but women had lower median white blood cell counts (9.5 k/cu mm versus 13.2 × 10^9^/L; *P* = 0.02). The median JAK2 V617F allele burden was higher in men compared to women (63% versus 53%; *P* = 0.05). There were no differences in the prevalence of MPN-directed therapies, including hydroxyurea (*P* = 0.28); anagrelide (*P* = 0.42); interferon (*P* = 1) between men and women (Table 1).

### 3.2. Vascular Complication History

In the whole cohort, 63 of 270 (23%) patients had a history of vascular complications; there were no statistical differences in rate of these complications between those with ET (13/61: 21%); PV (40/161: 25%); or MF (10/48: 21%). Of 164 women, 44 (27%) had a history of a vascular complication, compared to 19 of 106 men (18%); however, this difference was not statistically significant (*P* = 0.09). Ten patients had multiple vascular events, of which 7 were women. Among the 10 patients with multiple events, in most cases, different vascular beds were involved (Table 2). The median age at the time of vascular event did not differ by gender (56 years in men and women).

### 3.3. Type of Vascular Event

Among men, of 24 events, 10 (42%) were major venous events, 8 were minor events (33%), and 6 (25%) were major arterial events (25%). The most commonly affected vascular beds were cardiac (acute coronary syndromes in 5 men) or the deep venous system (deep vein thrombosis or pulmonary embolism in 5 men). Similarly, in women, of 51 events, 25 (49%) experienced major venous events, 15 (29%) had minor events, and 11 (22%) had major arterial vascular complications. However, in women, the most commonly affected vascular bed involved the abdominal venous system (AVT); 16 of 51 (31%) events involved the hepatic, portal, mesenteric, or splenic veins. Among women with AVT, most had PV (10/16: 63%), often involving the hepatic vein (Budd-Chiari syndrome). However, in women with MF, AVT involved the portal vein in 3 cases and was an associated complication of splenectomy in 2 of these cases. Transient ischemic attacks (8 events) and DVT/PE (8 events) represented the next most common type of vascular complication, followed by cerebrovascular accident (6 events). Chemotherapy or aspirin use at the time of the event did not differ by gender and was present in 9% and 18% of the cohort, respectively.

### 3.4. Timing of 1st Vascular Event

**Figure 1:**
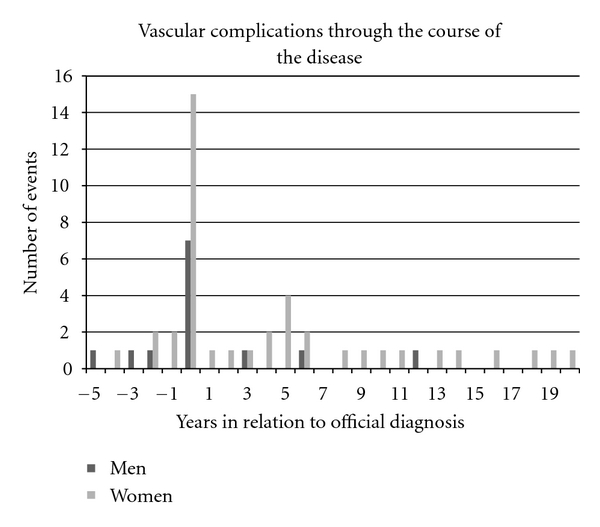
Major and minor vascular events through the course of the disease. The timing of vascular complication was known for 13 men and 41 women. In both men and women, vascular complications often lead to an official diagnosis of a myeloproliferative neoplasm (complication at year 0). Further, vascular complications predominantly occurred in the first decade of disease in both men and women. Of the 15 women with vascular complications at year 0, 9 had abdominal venous thrombosis.

The time of the first vascular event was known for 13 of 18 men (Figure 1). Among these, 12/13 (92%) had an event during the first decade of their disease. Ten (77%) either presented with a vascular complication (year 0 in Figure 1) that led to the official MPN diagnosis (7 men), or had an event in the preceding 5 years (3 men). Of the 7 men who presented with a vascular event leading to MPN diagnosis (year 0 in Figure 1), 1 (14%) had an abdominal venous thrombosis, 2 had DVT/PE, 2 had microvascular disturbances, and 2 had ACS. 

Among 44 women, the time of event in relation to diagnosis was known for 41 (Figure 1). Thirty-three of 41 (80%) women experienced a vascular complication during the first decade of disease. Twenty of 41 (49%) women either presented with a vascular complication that led to an official MPN diagnosis (15 women), or had an event in the preceding 5 years (5 women); this proportion was lower, but not statistically different when compared to men (49% versus 77%; *P* = 0.076), suggesting a trend among women who were more likely to experience a complication after an official diagnosis compared to men. The median time to a vascular event was comparable between men and women (0 (range −5 to 12) years versus 1.0 (range −4 to 20) years; *P* = 0.06). 

Of the 15 women who presented with a vascular event leading to an immediate MPN diagnosis (year 0 in Figure 1), 9 (60%) had abdominal venous thrombosis; 8 of these 9 women had PV. One woman was taking oral contraceptives (OCP) at presentation; 3 had no such history, and exposure was unknown in the remaining 5 women. Importantly, compared to men who presented with a vascular event, this predilection towards AVT at diagnosis was statistically significant (60% versus 14%; *P* = 0.04). Among the rest of the women with a vascular events at presentation, 2 had microvascular complications (both had PV), 2 had cardiac events (1 ET, 1 PV), 1 had a TIA (PV), and one had a brachial artery thrombosis (ET).

### 3.5. Multivariable Regression

In the univariate analysis (Table 1), there were gender differences in the whole cohort with regard to disease distribution, age at diagnosis, smoking history, dyslipidemia, median white blood cell count, and median JAK2 V617F allele burden. Therefore, these variables were incorporated in a multivariable logistic regression analysis (Table 3). When adjusting for these variables, women had an odds of a vascular event history that was 1.9 times higher compared to men (OR 1.89; 95% CI: 0.96, 3.73; *P* = 0.067). Among the above variables, dyslipidemia was marginally independently associated with a history of vascular complication (OR: 2.3; 95% CI: 1.0, 5.3; *P* = 0.045). For unknown reasons, those with a history of tobacco use were less likely to have a history of vascular complication (OR 0.48; 95% CI: 0.22, 1.0; *P* = 0.049), but this association has no biologic rationale, and for practical purposes, is insignificant.

## 4. Discussion

In this MPN cohort, women were younger at diagnosis, were less likely to have a history of tobacco use or dyslipidemia, and had lower median white blood cell counts and JAK2 V617 allele burdens, compared to men. Therefore, it was surprising to us that a history of a vascular event was at least as common in women, compared to men, with 27% of women experiencing vascular complications, compared to 18% of men (*P* = .09). In multivariable regression analysis, dyslipidemia associated with a history of a vascular event. In both men and women, the majority of vascular complications occurred in the 1st decade of the disease, similar to what has been observed by others in treated and untreated ET cases as well as PV cases [17–19]. In both men and women, a vascular complication often led to the diagnosis of an MPN. However, the type of event differed, more commonly involving the abdominal venous system in women ultimately diagnosed with PV. Therefore, when evaluating gender differences in phenotype, an important finding emerges: women manifest their MPN diagnosis differently, presenting with Budd-Chiari syndrome. 

Our association between disease presentation, female gender, JAK2-V617F positivity, and abdominal venous thrombosis has also been recognized by others. In a series of 93 patients with extrahepatic portal vein obstruction (EHPVO) or Budd-Chiari syndrome (BCS), approximately 60% were women (*n* = 56); this proportion was similar when looking at these entities separately [20]. Twelve percent of affected patients were taking oral contraceptives. The JAK2 V617F mutation was found in 36.5% of patients [20]. Colaizzo et al. observed 99 patients with portal/mesenteric vein thrombosis and identified a JAK2 V617F mutation in 17 patients [21]. Of these 17, 12 (70%) patients were women; identified risk factors were rare as only 1 woman was taking an oral contraceptive, 1 had hyperhomocysteinemia, and 1 had abdominal surgery [21]. In a series of 241 splanchnic vein thrombosis patients, Kiladjian et al. found JAK2 V617F mutations in 94. Among these 94, 58 were women [22]. The female predominance was particularly in those with BCS, and among 47 with JAK2 V617F mutations, 76% were women (*n* = 36) and most had PV. Prothrombotic factors, including factor V leiden mutations (19%), antiphospholipid antibodies (16%), and protein C deficiency (15%) were identified in those JAK2 V617F positive patients, but prevalence of OCP use was unknown [22]. More recently, Colaizzo et al. evaluated 180 patients with splanchnic vein thrombosis, with an aim of evaluating whether or not gender modulated the role of JAK2 V617F as a susceptibility factor for SVT [23]. In this series, among SVT patients, JAK2 V617F was found more frequently in women compared to men (OR 2.2; 95% CI 1.1–4.5). Further, the investigators found that the JAK2 46/1 haplotype associated with the occurrence of somatic JAK2 V617F in women, but not men. The authors speculated that the higher prevalence of JAK2 V617F in women with SVT might be accounted for by sex-related factors that contribute to mutation development, via the 46/1 haplotype [23].

Our study, like any other observational study, can only identify associations, rather than implying causal relationships. There remains a potential for residual confounding, as other unknown factors may account for gender differences described in this analysis. Further, oral contraceptive use was not known for many women, but is a risk factor for thrombosis, particularly of the abdominal venous system. However, the aforementioned series of splanchnic vein thrombosis, OCP use was documented only in the minority of JAK2 V617F-positive patients. Likewise, a hypercoagulable workup was not always done, or available in the medical record; in Kiladjian's series, nearly 40% of patients had identified abnormalities [22]. Lastly, limitations in power may impede recognition of true differences between men and women in our cohort.

Given the above, the mechanism behind the association between abdominal venous thrombosis and MPN remains unknown. Traditional factors that increase vascular risk in the MPN include age over 60 and prior thrombosis, and newly suggested risk factors include leukocytosis and increased JAK2 V617F allele burden [19, 24–26]. In our series, and that of others with abdominal venous thrombosis [22], leukocytosis was not prevalent, and JAK2 V617F allele burdens implied heterozygosity; therefore, a unique mechanism for thrombosis in this vascular bed exists, and is probably gender associated. 

Our analysis indicates that in the MPN, vascular risk is not easily attributed to either traditional population risk factors, or to MPN-specific, disease burden-related factors. Despite their younger age, less prevalent dyslipidemia or smoking history, lower white blood counts, and a lower JAK2 V617F allele burden, women had higher rates of AVT and had comparable rates of all other vascular complications. Our analysis contributes to a growing literature emphasizing gender differences in the MPN, which already includes differences in the JAK2 V617F allele burden and disease class and further supports the important impact of individual and host variation on the clinical manifestations of the MPN [27].

## Figures and Tables

**Table 1:** Baseline characteristics in the MPN cohort, by gender.

	Female	Male	*P* value
*Diagnosis, number (%)*			
* All MPN*	164	106	0.001
ET	43 (26)	18 (17)	
PV	104 (64)	57 (54)	
MF	17 (10)	31 (29)	

*Age at diagnosis, median (IQR)*			
* All MPN*	50 (37–61)	56 (48–67)	0.001
ET	50 (36–59)	48 (31–60)	
PV	48 (34–61)	56 (50–66)	
MF	58 (51–63)	61 (51–67)	

*Disease duration, median (IQR)*			
* All MPN*	5 (3–11)	5 (1–9)	0.04
ET	4 (2–10)	3 (1–9)	
PV	6 (3–12)	4 (1–8)	
MF	6 (3–12)	6 (2–11)	

*Hypertension, number (%)*	2 missing		
* All MPN*	53 (33%)	44 (42%)	0.15
ET	13	4	
PV	35	29	
MF	5	11	

*Diabetes Mellitus, number (%)*		1 missing	
* All MPN*	7 (4%)	8 (8%)	0.28
ET	0	0	
PV	5	6	
MF	2	2	

*Dyslipidemia, number (%)*			
* All MPN*	15 (9)	26 (25)	0.001
ET	2	3	
PV	12	15	
MF	1	8	

*Smoking history, number (%)*			
* All MPN*	35 (22)	40 (39)	0.005
ET	12	4	
PV	17	25	
MF	6	11	

*Median white blood cell count, × 10^9^/L (IQR)*			
* All MPN*	9.5 (7.3, 14.8)	13.2 (8.3, 21.4)	0.02
ET	8 (6.8, 9)	8.7 (6–10.8)	
PV	11.1 (7.5,18.4)	13.9 (9.6, 20)	
MF	25 (6.9, 49)	15.9 (7.9, 26.6)	

*Median hemoglobin, g/dL; (IQR)*			
* All MPN*	12.9 (11.6, 14)	13.2 (11.4, 14.4)	0.73
ET	13.7 (12.7, 14.2)	14.7 (13.2–15.1)	
PV	12.8 (11.6, 13.8)	13.6 (12.6–14.4)	
MF	10.6 (9.9, 12.5)	10 (9, 11.8)	

*Median Platelet count, × 10^9^/L (IQR)*			
* All MPN*	527 (333, 678)	450 (246–673)	0.09
ET	568 (499–782)	476 (412–654)	
PV	526 (340–667)	502 (300–700)	
MF	214 (133–330)	272 (166, 477)	

*JAK2 V617F allele burden, median (IQR)*			
* All MPN*	53 (42–69)	63 (46–83)	0.05
ET	34 (22–44)	44 (34–48)	
PV	64 (50–71)	71 (55–88)	
MF	82 (55–100)	57 (48–77)	

*JAK2 genotype*			0.1
Heterozygous	86 (53%)	45 (42%)	
Homozygous	77 (47%)	61 (58%)	

*Anti-platelet Use, number (%)*			
* All MPN*	81 (59)	69 (69)	0.12
ET	26	11	
PV	50	41	
MF	5	17	

*Warfarin Use, number (%)*			
* All MPN*	17 (10%)	7 (7%)	0.28
ET	4	3	
PV	13	4	
MF	0	0	

*Hydroxyurea Use, number (%)*			
* All MPN*	48 (30%)	28 (27%)	0.58
ET	11	3	
PV	33	17	
MF	4	8	

*Interferon Use, number (%)*			
* All MPN*	5 (3%)	3 (3%)	0.99
ET	0	1	
PV	4	0	
MF	1	2	

*Anagrelide Use, number (%)*			
* All MPN*	11 (7%)	4 (4%)	0.42
ET	4	1	
PV	6	1	
MF	1	2	

*7 women with missing data.*4 men with missing data.**27 women with missing data.**6 men with missing data.

**Table 2:** Major and minor vascular events, by gender.

	Women (*n* = 164)	Men (*n* = 106)	*P* value
Vascular event history, number (%)	44 (27)	19 (18)	0.09
ET	8 (18)	5 (26)	
PV	31 (71)	9 (47)	0.06
MF	5 (11)	5 (26)	

Vascular events, number	**51**	**24**	
Major Venous Events, number (%)	**25 (49)**	**10 (42)**	0.57
*DVT/PE *	**8 (15.7)**	**5 (20.8)**	0.47
ET	1	0	
PV	6	4	
MF	1	1	
*Abdominal venous thrombosis*	**16 (31.4)**	**4 (16.7)**	0.26
ET	2	2	
PV	10	1	
MF	4	1	
*Sinus venous thrombosis*	**1 (2)**	**1 (4.2)**	0.61
ET	0	1	
PV	1	0	
MF	0	0	
Major Arterial Events, number (%)	**11 (22)**	**6 (25)**	0.77
*Cardiac (ACS) *	**5 (9.8)**	**5 (20.8)**	0.14
ET	3	1	
PV	2	3	
MF	0	1	
*Neurologic (CVA)*	**6 (11.8)**	**1 (4.2)**	0.31
ET	1	0	
PV	5	1	
MF	0	0	
Minor events, number (%)	**15 (29)**	**8 (33)**	0.73
*TIA *	**8 (15.7)**	**3 (12.5)**	0.81
ET	1	0	
PV	7	1	
MF	0	2	
*Peripheral arterial thrombosis*	**3 (5.8)**	**2 (8.3)**	0.6
ET	1	0	
PV	1	1	
MF	1	1	
*Microvascular disturbance*	**4 (7.8)**	**3 (12.5)**	0.42
ET	2	2	
PV	2	1	
MF	0	0	

**Table 3:** Multivariable logistic regression.

Vascular event history	Odds ratio	*P* value	95% CI: lower limit	95% CI: upper limit
Female sex	1.89	0.07	0.96	3.73
PV compared to ET	1.06	0.89	.44	2.53
MF compared to ET	1.1	0.87	.35	3.42
Age at diagnosis	.99	0.61	.97	1.01
Dyslipidemia	2.31	0.05	1.0	5.34
Smoking history	.48	0.049	.23	1.0
White Blood Cell count	1.0	0.66	.98	1.03
JAK2 V617F allele burden	1.0	0.77	.98	1.02
